# Supplementary material for: The Magnitude of NCD Risk Factors in Ethiopia: Meta-Analysis and Systematic Review of Evidence
Source: Int J Environ Res Public Health. 2022 Apr 27;19(9):5316. doi: 10.3390/ijerph19095316 (PMC9106049; doi:10.3390/ijerph19095316)
Supplement: Supplementary file 1 [file ijerph-19-05316-s001.zip › Supplementary Table S1.pdf]

Supplementary table 1: Shows the characteristics and quality assessment score of studies on hypertension.

**Authors name and year**	**Region**	**Sample size**	**Prevalence (%)**	**Quality Score**
Abebe, et al. (2015)	Amhara	2141.00	27.9	10
Abebe, et al 2017	Amhara	67397.000	27.7	10
Alemseged, et al.2012	Oromia	5000.00	9.3	7
Anteneh, et al. (2015).	Amhara	681.00	25.1	10
Asfaw, et al. (2018)	SNNP	525.00	30	9
Asresahegn, (2017)	Somali	492.00	28.3	8
Awoke, et al. (2012)	Amhara	679.00	28.3	10
Bekele,et al. (2017)	Ethiopia	10260.00	15	10
Bonsa, et al. (2014)	Oromia	384.00	23.8	6
Demisse, et al. (2017)	Amhara	3227.00	27. 4	10
Desalegn, H., et al. (2017)	Oromia	576.00	23.8	7
Gebremariam, et al. (2018)	Tigray	1500.00	18.85	6
Gebreyes, et al. (2018)	Ethiopia	10260.00	15.8	10
Geleta, et al. (2019)	Oromia	711.00	34.9	10
Fikadu and Lemma (2016)	Addis Ababa	1866.00	16.9	8
Hailemichael et al. (2017).	Oromia	576.00	23.8	4
Helelo, et al. (2014)	SNNP	518.00	22.4	10
Hassen and Mamo (2019)	Amhara	318.00	30.7	10
Kebede, et al.2020	SNNP	784.00	13.2	10
Kiber, et al (2019)	Amhara	477.00	12.5	10
Mengistu, (2014)	Tigray	1183.00	18.1	10
Moges, et al. (2014)	Amhara	68.00	13.3	4
Roba, et al. (2019).	Diredawa	903.00	24.4	9
Seifu, et al. (2016).	Afar	548.00	7.9	6
Shukuri, et al. (2019)	Oromia	702	41.9	10
Tesfaye, et al. (2019)	Amhara	1405	13.0	10
Zekewos, et al. (2019)	SNNP	1952	21.8	8
