# Supplementary material for: The Magnitude of NCD Risk Factors in Ethiopia: Meta-Analysis and Systematic Review of Evidence
Source: Int J Environ Res Public Health. 2022 Apr 27;19(9):5316. doi: 10.3390/ijerph19095316 (PMC9106049; doi:10.3390/ijerph19095316)
Supplement: Supplementary file 1 [file ijerph-19-05316-s001.zip › Supplementary Table S2.pdf]

**Supplementary table 2**: Shows the characteristics and quality assessment of score of studies related to overweight.

| Authors name and year | Region | Sample size | Prevalence (%) | Quality score |
|---|---|---|---|---|
| Abrha, et al. (2016) | Ethiopia | 3602.00 | 12.1 | 10 |
| Dagne, et al. (2019) | Amhara | 751.00 | 19.9 | 10 |
| Darebo et al. (2019) | SNNP | 531.00 | 23 | 10 |
| Mekonnen, et al. (2018) | Amhara | 1484.00 | 9.3 | 10 |
| Moges, et al. (2014) | Amhara | 68.00 | 32.4 | 4 |
| Seifu, et al. (2016). | Oromia | 548.00 | 8.8 | 6 |
| Yohannes, (2019). | SNNP | 374.00 | 40.1 | 8 |
