# Supplementary material for: The Magnitude of NCD Risk Factors in Ethiopia: Meta-Analysis and Systematic Review of Evidence
Source: Int J Environ Res Public Health. 2022 Apr 27;19(9):5316. doi: 10.3390/ijerph19095316 (PMC9106049; doi:10.3390/ijerph19095316)
Supplement: Supplementary file 1 [file ijerph-19-05316-s001.zip › Supplementary Table S3.pdf]

Supplementary table 3: Shows the characteristics and quality assessment score of studies related to obesity.

Author's name and year	Region	Sample size	Prevalence (%)	Quality score
Abrha, et al. (2016)	Ethiopia	3602.00	2.8	10
Dagne, et al. (2019)	Amhara	751.00	8.6	10
Darebo et al. (2019)	SNNP	531.00	5	10
Mekonnen, et al. (2018)	Amhara	1484.00	2	10
Moges, et al. (2014)	Amhara	68.00	16.2	4
Seifu, et al. (2016).	Oromia	548.00	38.5	6
Yohannes, (2019).	SNNP	374.00	13.9	8
