# Supplementary material for: The Magnitude of NCD Risk Factors in Ethiopia: Meta-Analysis and Systematic Review of Evidence
Source: Int J Environ Res Public Health. 2022 Apr 27;19(9):5316. doi: 10.3390/ijerph19095316 (PMC9106049; doi:10.3390/ijerph19095316)
Supplement: Supplementary file 1 [file ijerph-19-05316-s001.zip › Supplementary Table S4.pdf]

Supplementary table 4: Shows the characteristics and quality assessment score of studies related to Physical activity.

Author's name and year	Region	Sample size	Prevalence (%)	Quality score
Alemseged, et al. (2012)	Oromia	5,000	83.1	7
Amenu, K., et al. (2017).	National	10260	94.2	10
Kassahun, et al. (2017)	National	10260	94.2	10
Mengesha, et al. (2019)	Dire Dawa	903	65.9	10
Seifu, et al. (2016).	Afar	548	18.1	6
