# Supplementary material for: The Magnitude of NCD Risk Factors in Ethiopia: Meta-Analysis and Systematic Review of Evidence
Source: Int J Environ Res Public Health. 2022 Apr 27;19(9):5316. doi: 10.3390/ijerph19095316 (PMC9106049; doi:10.3390/ijerph19095316)
Supplement: Supplementary file 1 [file ijerph-19-05316-s001.zip › Supplementary Table S5.pdf]

Supplementary table 5: Shows the characteristics and quality assessment score of studies related to smoking.

Author's name and year	Region	Sample size	Data collection technique	Prevalence of all types of smoking (%)	Quality score
Abebe, et al 2017	Amhara	67 397		never smoked	10
Alemseged, et al.2012	Oromia	5,000		9.3	7
Eticha and Kidane (2014).	Tigray	193		29.5%	7
Guliani, et al. (2019)	national	56, 644		3.21	10
Lakew and Haile (2015)	national	30,625		4.1	10
Petersen, et al. (2016).	SNNP	353		1	10
Reda, et al. (2013)	Oromia	548		38.6	10
Seifu, et al. (2016).	Afar	548		13.5	6
Sinba Etu, et al. (2017)	Oromia	634		45.3%	10
