# Supplementary material for: The Magnitude of NCD Risk Factors in Ethiopia: Meta-Analysis and Systematic Review of Evidence
Source: Int J Environ Res Public Health. 2022 Apr 27;19(9):5316. doi: 10.3390/ijerph19095316 (PMC9106049; doi:10.3390/ijerph19095316)
Supplement: Supplementary file 1 [file ijerph-19-05316-s001.zip › Supplementary Table S6.pdf]

Supplementary table 6: Shows the characteristics and quality assessment score of studies related to alcohol.

Author's name and year	Region	Sample size	Prevalence (%)	Quality score
Abebe, et al 2017	Amhara	67 397	7.1	10
Alemseged, et al.2012	Oromia	5,000	65.4	10
Bhagavathula, A. S., et al. (2017)	SNNP	400	12.4	6
Getachew, et al. (2017)	national	10,260	13.5	10
Seifu, et al. (2016).	national	548	24.6	6
Tegegn, (2013)	Not mentioned	861	21.1	10
Teferra, et al. (2014)	Not mentioned	1 500		10
