# Supplementary material for: The Magnitude of NCD Risk Factors in Ethiopia: Meta-Analysis and Systematic Review of Evidence
Source: Int J Environ Res Public Health. 2022 Apr 27;19(9):5316. doi: 10.3390/ijerph19095316 (PMC9106049; doi:10.3390/ijerph19095316)
Supplement: Supplementary file 1 [file ijerph-19-05316-s001.zip › Supplementary Table S7.pdf]

Supplementary table 7: Shows the characteristics and quality score of studies related to metabolic syndrome.

Authors name and year	Region	Sample size	Measure	Prevalence (%)	Quality score
Alemseged, et al.2012	Oromia	5,000	High cholesterol level	10.7	7
			High triglycerides	7.7	
Gebreyes, et al. (2018)	national	10,260	Metabolic syndrome	4.8	10
			Hypercholesterolemia	5.2	
Hailemichael et al. (2017).	Oromia	576	Prevalence of high cholesterol	5.5	4
Kerie, et al. (2019)	Oromia	558	Prevalence of metabolic syndrome	9.6	9
Seifu, et al. (2016).	Afar	548	High triglycerides	68.7%	6
