# Supplementary material for: The Magnitude of NCD Risk Factors in Ethiopia: Meta-Analysis and Systematic Review of Evidence
Source: Int J Environ Res Public Health. 2022 Apr 27;19(9):5316. doi: 10.3390/ijerph19095316 (PMC9106049; doi:10.3390/ijerph19095316)
Supplement: Supplementary file 1 [file ijerph-19-05316-s001.zip › Supplementary Table S8.pdf]

Table 8: Shows the characteristics and quality assessment of studies related to fruit consumption.

Author's name and year	Region	Sample size	Prevalence (%)	Quality score
Hailemichael et al. (2017).	Oromia	576<br>548	Universal	
Seifu, et al. (2016).	Afar	548	97.9%	
